# Pulsatile gonadotropin releasing hormone therapy for spermatogenesis in congenital hypogonadotropic hypogonadism patients who had poor response to combined gonadotropin therapy

**DOI:** 10.20945/2359-4292-2023-0101

**Published:** 2024-05-10

**Authors:** Zhenxing Huang, Xi Wang, Bingqing Yu, Wanlu Ma, Pengyu Zhang, Xueyan Wu, Min Nie, Jiangfeng Mao

**Affiliations:** 1 Peking Union Medical College Chinese Academy of Medical Sciences Peking Union Medical College Hospital Beijing China Department of Endocrinology, Peking Union Medical College Hospital, Chinese Academy of Medical Sciences, Peking Union Medical College, Beijing, China; 2 The First Affiliated Hospital of Guangxi Medical University Department of Endocrinology Nanning China Department of Endocrinology, The First Affiliated Hospital of Guangxi Medical University, Nanning China; 3 The First Affiliated Hospital of Zhengzhou University Department of Endocrinology Zhengzhou China Department of Endocrinology, The First Affiliated Hospital of Zhengzhou University, Zhengzhou, China

**Keywords:** Congenital hypogonadotropic hypogonadism, gonadotropin therapy, pulsatile gonadotropin-releasing hormone therapy, spermatogenesis

## Abstract

**Objective:**

Both pulsatile gonadotropin-releasing hormone (GnRH) and combined gonadotropin therapy are effective to induce spermatogenesis in men with congenital hypogonadotropic hypogonadism (CHH). This study aimed to evaluate the effect of pulsatile GnRH therapy on spermatogenesis in male patients with CHH who had poor response to combined gonadotropin therapy.

**Materials and methods:**

Patients who had poor response to combined gonadotropin therapy ≥ 6 months were recruited and shifted to pulsatile GnRH therapy. The rate of successful spermatogenesis, the median time to achieve spermatogenesis, serum gonadotropins, testosterone, and testicular volume were used for data analysis.

**Results:**

A total of 28 CHH patients who had poor response to combined gonadotropin (HCG/HMG) therapy for 12.5 (6.0, 17.75) months were recruited and switched to pulsatile GnRH therapy for 10.0 (7.25, 16.0) months. Sperm was detected in 17/28 patients (60.7%). The mean time for the appearance of sperm in semen was 12.0 (7.5, 17.5) months. Compared to those who could not achieve spermatogenesis during pulsatile GnRH therapy, the successful group had a higher level of LH_60min_ (4.32 vs. 1.10 IU/L, P = 0.043) and FSH_60min_ (4.28 vs. 1.90 IU/L, P = 0.021). Testicular size increased during pulsatile GnRH therapy, compared to previous HCG/HMG therapy (P < 0.05).

**Conclusion:**

For CHH patients with prior poor response to one year of HCG/HMG therapy, switching to pulsatile GnRH therapy may induce spermatogenesis.

## INTRODUCTION

Congenital hypogonadotropic hypogonadism (CHH) is a rare disorder caused by a deficiency of gonadotropin releasing hormone (GnRH) (1). The incidence of CHH is approximately 1 in 10,000 amongst the general population (2), and patients are classified into two categories according to olfactory status. Those presenting anosmia are considered to have Kallmann syndrome (KS) and others with normal olfactory function are defined as normosmic congenital hypogonadotropic hypogonadism (nCHH) (3).

Most CHH cases are treatable regarding infertility. Both pulsatile GnRH therapy and combined gonadotropin (human chorionic gonadotropin/human menopausal gonadotropin, HCG/HMG) therapy are effective in inducing spermatogenesis in 60%-85% patients (4,5). Clinical studies and meta-analysis have reported near equivalent outcomes to fertility induction via GnRH and combined gonadotropins (6-8), while the latter treatment is much cheaper and more available in some countries. Given the cost-effectiveness and availability, HCG/HMG therapy is more commonly used in most medical centers (9-11).

The question remains on how to deal with CHH patients who respond poorly to HCG/HMG therapy. It is yet unknown if the people with poor outcome would respond to subsequent pulsatile GnRH therapy. Although some case reports have hinted that switching to pulsatile GnRH therapy may induce spermatogenesis (12,13), the general effects on spermatogenesis could not be determined due to the small number of the patients examined. Therefore, the purpose of this study is to investigate the effects of pulsatile GnRH therapy on patients who previously had poor response to HCG/HMG therapy.

## MATERIALS AND METHODS

### Ethics statement

This study was approved by the Ethics Review Committee of Peking Union Medical College Hospital (No.JS-2111). Written informed consent was obtained from all participants.

### Participants

**Figure 1:**
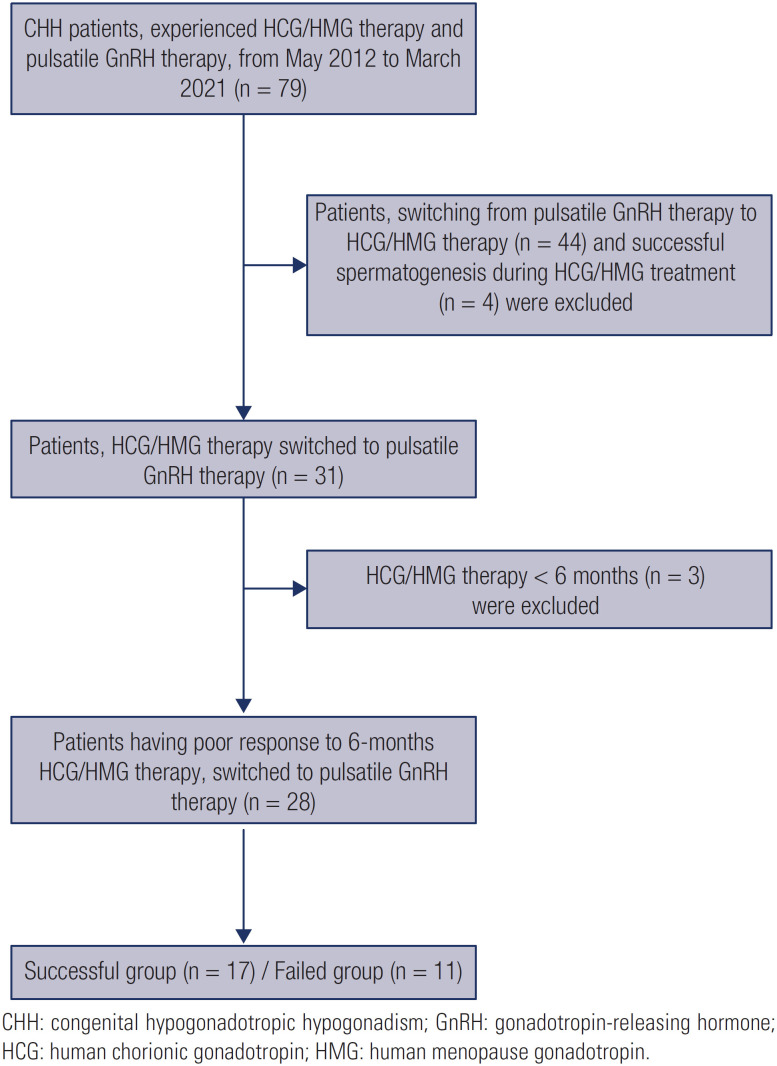
Flow chart for patient recruit.

There were 79 CHH patients who had experienced HCG/HMG therapy and pulsatile GnRH therapy, between May 2012 and March 2021, in Department of Endocrinology. Among them, 28 male patients were eligible according to our inclusive criteria (poor response to HCG/HMG therapy) and were enrolled in this retrospective clinical trial. CHH was diagnosed according to the criteria reported previously (14). The inclusion criteria were as follows: (1) patient had experienced HCG/HMG therapy for ≥ 6 months and azoospermia during HCG/HMG therapy; (2) Increment of testicular size < 4 mL, or serum testosterone < 1.5 ng/mL during HCG/HMG therapy; and (3) switched to pulsatile GnRH therapy for ≥ 6 months. The exclusion criteria were (1) acquired HH; (2) patients received HCG/HMG < 6 months; (3) appearance of sperm in semen during HCG/HMG therapy. The flow chart for patient recruitment is listed in Figure 1.

### Clinical and laboratory data

Testicular volume from descended testis or after orchidopexy, was measured by Prader orchidometer. For undescended testes, the testis volume was defined as 1 mL. The mean value of bilateral testicular volume was used for data analysis. The levels of testosterone, luteinizing hormone (LH) and follicle stimulating hormone (FSH) were measured at the central laboratory of the hospital with a chemiluminescent immunoassay. Seminal analysis was performed according to World Health Organization guidelines (fifth version) (15). Triptorelin stimulation test was performed before pulsatile GnRH therapy. Triptorelin (100 μg) was injected intramuscularly, and serum LH and FSH were measured immediately and 60 min after injection. In addition, a gene panel including 31 CHH-related genes was used to capture gene mutations following the instructions of previously published paper (16).

### Interventions and follow-up

HCG/HMG therapy: Combined HCG (2000-5000 U, Livzon Pharmaceutical Co., Guangdong, China) and HMG (75-150 U, Livzon Pharmaceutical Co.) was intramuscularly injected twice weekly. Gonadotropin dosages were adjusted in order to maintain serum testosterone levels within 2.5-5 ng/mL.

Pulsatile GnRH therapy: Pulsatile GnRH (Fengyuan Pharmaceutical Co., Anhui Province, China) was subcutaneously administered via a portable infusion pump (Weichuang Medical Science Co., Shanghai, China) for at least 6 months. The starting dosage was 10 μg per 90 minutes, which was adjusted to attain LH and FSH levels between 3-10 IU/L.

Regular follow-up was conducted at intervals of 2-3 months during the therapy. Testicular size, serum levels of testosterone, LH, FSH and sperm count were measured on each visit. Patients lost to follow-up were not included in this study.

### Outcomes

The primary outcome was the time of successful spermatogenesis, which was defined as the observation of one sperm by microscope in centrifuged ejaculate fluid. Other outcomes included the testicular size and serum testosterone levels.

### Statistical analyses

SPSS version 20.0 (IBM Corp., Armonk, NY, USA) was used for data analysis. Normally distributed data were presented as mean ± standard deviation (s.d.), non-normally distributed data were expressed as medians (quartiles), and categorical variables were listed as numbers (percentages). Comparison between groups was carried out using the unpaired t-test and Chi-squared test. The paired samples t-test was performed to analyze differences in testicular size and the peak serum testosterone level during pulsatile GnRH and gonadotropin therapy. Logistic regression analysis was used to examine the potential factors influencing spermatogenesis. Missing data were not substituted with estimated values. Statistical significance was set at *P* < 0.05.

## RESULTS

### Characteristics of Patients with CHH

A total of 28 male CHH patients (10 [35.7%] with nCHH and 18 [64.3%] with KS) were retrospectively evaluated in this study. The demographic and clinical characteristics of the patients are presented in Table 1. The durations of HCG/HMG therapy and pulsatile GnRH therapy were 12.5 (6.0, 17.75) and 10.0 (7.25, 16.0) months, respectively. The number of patients treated with HCG/HMG for 6-11 months, 12-17 months, 18-23 months, and more than 24 months were 11, 10, 3, 4, respectively. The interval time between two treatments were 4.5 months. The general spermatogenesis rate was 60.7% (17/28) following pulsatile GnRH therapy. Patients were divided into two groups (successful and failed) based on the presence of sperm. The time of pulsatile GnRH therapy was similar between the two groups. No serious adverse events occurred. There was no significant difference in diagnosis, age, BMI, cryptorchidism, history of testosterone replacement therapy, baseline serum LH, FSH, treatment interval time, or testosterone. The successful group had a higher LH level and larger testicular size than the failed group during GnRH therapy (t = 4.312, P < 0.001, t = 2.659, P < 0.05, respectively).

**Table 1:** Comparison between two groups according to sperm outcomes

		Total patients with CHH (n = 28)	Successful group (n = 17)	Failed group (n = 11)	P value
Diagnosis					
	nCHH, *n*(%)	10 (35.7%)	6 (35.3%)	4 (36.4%)	
	Kallmann syndrome, *n*(%)	18 (64.3%)	11 (64.7%)	7 (63.6%)	0.954
Age of starting GnRH therapy (yrs)		24.00 ± 4.80	24.65 ± 5.21	23.00 ± 4.12	0.385
BMI (kg/m^2^)		24.37 ± 4.20	25.32 ± 4.90	22.89 ± 2.29	0.090
Previous TRT, n (%)		19 (67.9%)	10 (58.8%)	9 (81.8%)	0.249
Cryptorchidism, n (%)		7 (25.0%)	6 (35.3%)	1 (9.1%)	0.191
Baseline					
	Basal testicular size (mL)	1.68 ± 0.63	1.82 ± 0.66	1.45 ± 0.52	0.131
	Basal LH (IU/L)	0.26 ± 0.12	0.23 ± 0.06	0.32 ± 0.16	0.099
	Basal FSH (IU/L)	0.55 ± 0.37	0.53 ± 0.42	0.60 ± 0.30	0.638
	Basal testosterone (ng/mL)	0.39 ± 0.33	0.38 ± 0.32	0.40 ± 0.36	0.853
During HCG/HMG therapy					
	Duration of HCG/HMG therapy (Mons)	12.5 (6.0, 17.75)	13.0 (6.5, 19.0)	8.0 (6.0, 18.0)	0.363
	Testicular size (mL)	3.93 ± 2.24	4.41 ± 2.60	3.18 ± 1.33	0.160
	Testosterone (ng/mL)	2.33 ± 1.82	2.51 ± 2.10	2.05 ± 1.31	0.515
LH _60min_ (IU/L)		3.09 ± 4.26	4.32 ± 5.02	1.10 ± 1.16	0.043*
FSH _60min_ (IU/L)		3.33 ± 2.59	4.28 ± 2.85	1.90 ± 1.24	0.021*
During pulsatile GnRH therapy					
	Duration of GnRH therapy (mons)	10.0 (7.25, 16.0)	12.0 (7.5, 17.5)	9.0 (2.0, 12.0)	0.179
	Testicular size (mL)	8.45 ± 4.03	10.50 ± 3.33	5.27 ± 2.80	<0.001*
	LH (IU/L)	6.79 ± 5.88	8.94 ± 6.28	3.47 ± 3.21	0.013*
	FSH (IU/L)	9.36 ± 7.28	9.92 ± 6.34	8.49 ± 8.80	0.619
	Testosterone (ng/mL)	2.39 ± 1.73	2.90 ± 1.60	1.61 ± 1.69	0.051

CHH: congenital hypogonadotropic hypogonadism; BMI: body mass index; TRT: testosterone replacement therapy; LH: luteinizing hormone; FSH: follicle-stimulating hormone; LH _60min_: 60 minutes LH levels after stimulation by triptorelin 100 mg; FSH _60min_: 60 minutes FSH levels after stimulation by triptorelin 100 mg.

* *P* < 0.05 is defined as statistical significance.

### Induction of spermatogenesis

The successful group included 6 nCHH patients and 11 KS patients. The median time needed for spermatogenesis was 12.0 (7.5, 17.5) months during GnRH therapy and the sperm concentration was 6.32 ± 4.61 million/mL. There were 2, 3, 9, 13 and 15 patients who successfully produced sperm within 3, 6, 12, 18 and 24 months, respectively.

### Comparison of peak testosterone levels and testicular size during spermatogenesis treatment

**Figure 2:**
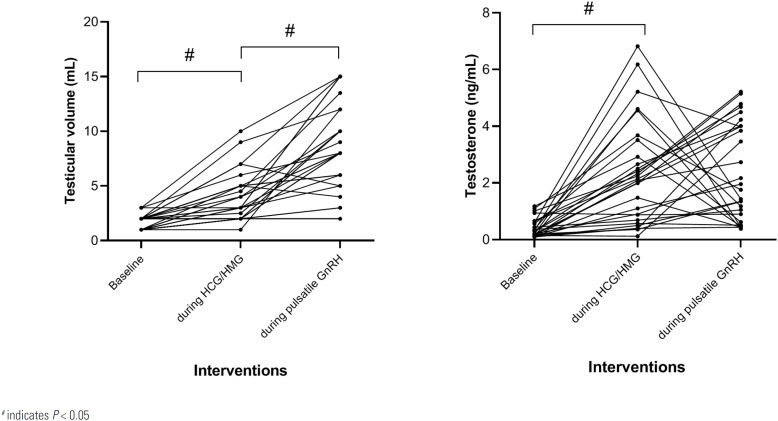
Change of serum Testosterone and testicular size during HCG/HMG and GnRH therapy

The resulting trends in testosterone levels and testicular size during HCG/HMG and GnRH interventions are presented in Figure 2. During HCG/HMG therapy, serum testosterone increased from 0.39 ± 0.33 to 2.33 ± 1.82 ng/mL (t = -5.795, P < 0.001) and testicular size increased from 1.68 ± 0.63 to 3.93 ± 2.24 mL (t = -5.788, P < 0.001). After shifting to pulsatile GnRH therapy, the peak testosterone level was maintained at 2.39 ± 1.73 ng/mL (t = -0.127, P = 0.90, compared to before GnRH therapy), and testicular size further increased to 8.45 ± 4.03 mL (t = -6.152, P < 0.001, compared to before GnRH therapy). These findings may indicate that pulsatile GnRH was more powerful in increasing testicular size than HCG/HMG therapy.

### Predictive factors for spermatogenesis during GnRH therapy

Compared with the failed group, the successful group had a higher level of LH_60min_(4.32 *vs.* 1.10 IU/L, t = 2.221, *P* = 0.043) and FSH_60min_ (4.28 *vs.* 1.90 IU/L, t = 2.555, *P* = 0.021) (Table 1). Binary logistic analysis (including 28 participants) determined that the basal testosterone concentration (*P* = -0.741), basal testicular size (*P*= 0.085), LH_60min_ (*P* = 0.141), FSH_60min_ (*P*= 0.549), as well as testicular size during HCG/HMG therapy (*P* = -0.265), were not significant predictors for spermatogenic outcome (Table 2).

**Table 2:** Predictors for Spermatogenesis during Pulsatile GnRH Therapy (Binary Logistic Analysis)

Factor	β	*P*value	95% CI lower bound	95% CI upper bound
Basal testosterone	-0.741	0.642	0.021	10.814
Basal testicular size	0.085	0.929	0.168	7.067
LH _60min_	0.141	0.663	0.610	2.172
FSH _60min_	0.549	0.269	0.654	4.588
Testicular size during HCG/HMG therapy	-0.265	0.545	0.326	1.807

LH _60min_ and FSH _60 min_: levels of LH and FSH after stimulated by triptorelin 100 ug, subcutaneously injected.

### Genetic screening

Of the 28 CHH patients, 8 patients (8/28, 28.6%) underwent targeted next-generation sequencing. Gene mutations in *FGFR1, PROKR2, FGF8*, and *CHD7* were detected in 7 patients (Table 3). The relationship between gene mutations and spermatogenesis could not be determined due to the limited number of patients.

**Table 3:** Genetic screening in 28 patients with CHH

	Not measured	No mutation detected	*CHD7*	*FGF8*	*FGFR1*	*PROKR2*	Total
Successful Group	9	1	1	0	1	1	13
Failed Group	11	0	0	1	3	0	15
Total	20	1	1	1	4	1	28

## DISCUSSION

This retrospective study showed that patients who failed to respond to 1 year of HCG/HMG therapy may benefit from switching to pulsatile GnRH therapy, and that production of sperm could be successfully induced in 60.7% (17/28) of CHH patients using this intervention. In addition, pulsatile GnRH therapy appears to induce larger testicular volume than HCG/HMG therapy (8.45 *vs.* 3.93 mL, *P* < 0.001). This is the first study to investigate the effects of subsequent GnRH therapy for patients who failed to respond to HCG/HMG therapy.

It is important to address why some patients had a poor response to HCG/HMG therapy and the following reasons should be considered. First, this group may not have received enough or appropriated HCG/HMH treatment. Second, the serum concentration of HCG was much higher than the normal physiological concentration of LH, which may over stimulate Leydig cell proliferation and testosterone production (17). Third, autoimmune antibodies to HCG in the serum may neutralize the effects of HCG (18,19). Finally, a high concentration of HCG may completely deplete LH receptors on Leydig cells (20,21).

Next, we must examine why pulsatile GnRH therapy improves the spermatogenesis outcome in this population. GnRH therapy may produce physiological and pulsatile gonadotropin secretion, thereby restoring the function of the pituitary gonadal axis (22). Another study observed that the GnRH receptor was expressed in human spermatogenic cells and mature spermatozoa (23), indicating that GnRH may directly promote spermatogenesis and testicular maturation. Several studies have reported that pulsatile GnRH therapy seemed to be superior to HCG/HMG therapy, with success rates ranging from 53.0% to 90.0% (10,11,24,25). Physiological and pulsatile LH and FSH fluctuation in response to GnRH stimulation may promote better testicular development, which was confirmed by the observation of larger testicular sizes during GnRH therapy (26). In addition, a randomized, open label study described a sequential hormone protocol in which recombinant FSH (rFSH) pretreatment was given prior to rFSH + HCG or pulsatile GnRH therapy (27). This sequential treatment showed superiority in inducing testicular growth and fertility in men with CHH (27). However, whether combined gonadotropin pretreatment followed by GnRH therapy have similar effects on maximizing the potential for fertility are still not clearly outlined.

Treatment duration is also an important factor for spermatogenesis, and inadequate treatment time may contribute to failure of spermatogenesis. Earlier studies have reported that the median time for the first detection of sperm was 9-18 months in patients receiving HCG/HMG therapy (9-11,28,29). Notably, the median time to achieve first sperm with pulsatile GnRH therapy was only 6.0-6.4 months (9-11). In the present study, the duration of HCG/HMG therapy and pulsatile GnRH therapy was 12.5 and 10 months respectively, and the time for spermatogenesis was 12 months after shifting to pulsatile GnRH therapy. For patients with poor response to HCG/HMG therapy over 12 months, prolonging treatment time is one of the options. A higher rate of spermatogenesis may be achieved if additional treatment time was given. Kobori and cols. reported that the effect of hCG + rhFSH on spermatogenesis was better than hCG alone or hCG + hMG in idiopathic HH patients (30). Therefore, changing the treatment medication may also work. Our results showed that alternating to GnRH therapy may induced spermatogenesis in 60% patients. Based on our study findings, we propose that for CHH patients who display a poor response to 12 months of HCG/HMG treatment, the alternative of pulsatile GnRH could be a reasonable option.

It has been suggested that genetic heterogeneity is linked to the response to spermatogenic treatment in CHH (31,32). More than 30 genes have been implicated in CHH (32). Chen and cols. suggested that *PROKR2* gene mutations might correlate with poor responses to HCG/HMG therapy in patients with CHH (33). A retrospective study identified, that *FGFR1* gene mutations may result in more severe gonadal axis defects and longer times for sperm production (34). In addition, *KAL1* mutations may impair testicular development and spermatogenesis (35). Another study reported that patients with *PROKR2* mutation exhibited a better response to gonadotropin and pulsatile GnRH therapy than the men with *FGFR1* mutations (36). Therefore, genetic mutations should be considered upon the evaluation of therapeutic effect. Only 8 patients (28.6%) completed genetic screening in this retrospective study and the relationship between gene mutations and the response to pulsatile GnRH therapy could not be clarified.

Logistic analysis was used to investigate other factors, such as cryptorchidism (37,38), testis volume (39,40), and stimulated levels of LH (41). The results of which showed that none of were affiliated with spermatogenesis outcome. These results are not statistically significant, likely due to the small sample size. Moreover, CHH is a clinically and genetically heterogeneous disorder, which may be a reason for the inconsistent findings in previous studies. Therefore, combination of phenotype and genotype could more accurately predict the prognosis of patients and guide treatments.

There were clear limitations to our work. First, studying a rare disease like CHH is not easy to control numerous variables, methodological limitations may bring clinical uncertainty. Second, treatment adherence was uncontrolled in this retrospective study, which may directly influence the outcome. Third, the number of cases completed gene screening was too small to analyze genetic heterogeneity. Last, the number of men included in this retrospective study was limited, a larger, prospective and randomized study is required to confirm our finding. However, the sample size of our study has been larger than related previous studies to make such a comparison.

In conclusion, for CHH patients who did not respond to 1 year of HCG/HMG therapy, switching to pulsatile GnRH may induce successful spermatogenesis in over 60% of patients. Our study provides a reference for clinical decision-making in the management of male CHH.

## Data Availability

the datasets generated to support the findings of this study are available from the corresponding author upon reasonable request.
